# Ensuring Confidentiality of Geocoded Health Data: Assessing Geographic Masking Strategies for Individual-Level Data

**DOI:** 10.1155/2014/567049

**Published:** 2014-04-29

**Authors:** Paul A. Zandbergen

**Affiliations:** Department of Geography, University of New Mexico, Albuquerque, NM 87131, USA

## Abstract

Public health datasets increasingly use geographic identifiers such as an individual's address. Geocoding these addresses often provides new insights since it becomes possible to examine spatial patterns and associations. Address information is typically considered confidential and is therefore not released or shared with others. Publishing maps with the locations of individuals, however, may also breach confidentiality since addresses and associated identities can be discovered through reverse geocoding. One commonly used technique to protect confidentiality when releasing individual-level geocoded data is geographic masking. This typically consists of applying a certain amount of random perturbation in a systematic manner to reduce the risk of reidentification. A number of geographic masking techniques have been developed as well as methods to quantity the risk of reidentification associated with a particular masking method. This paper presents a review of the current state-of-the-art in geographic masking, summarizing the various methods and their strengths and weaknesses. Despite recent progress, no universally accepted or endorsed geographic masking technique has emerged. Researchers on the other hand are publishing maps using geographic masking of confidential locations. Any researcher publishing such maps is advised to become familiar with the different masking techniques available and their associated reidentification risks.

## 1. Introduction

The widespread availability of powerful geocoding tools in commercial Geographic Information System (GIS) software and the interest in spatial analysis at the individual level have made mapping residential addresses of individuals a widely employed technique in public health research [1–6]. Spatial analysis and mapping of georeferenced, individual-level health data can help identify important geographical patterns [1, 2, 7, 8]. However, given the need and/or legal requirement to preserve the confidentiality of microdata, the possibilities of undertaking geographical analysis on certain types of individual-level data are often limited [9, 10]. As a result of restrictions on access to confidential data, important information may remain inaccessible.

Releasing locations of individuals in digital or paper format presents reidentifications risk since these locations can be reverse geocoded to find the addresses and identities associated with those locations. Geographic masking techniques have been developed to reduce the risk of reidentification. The present review describes the background for sharing and individual-level data, the use of geocoding and reverse geocoding of health-related datasets, and the effectiveness of geographic masking techniques to preserve confidentiality.

## 2. Individual-Level Data and Geocoding

Datasets collected as part of public health research often contain confidential information. This may include the individual's name, gender, age, race, ethnicity, income, and other socioeconomic characteristics, as well as the specific health-related conditions of interest in the particular study. The collection of this type of individual information for research purposes falls under human subjects' research. This type of data cannot be released publicly since this would violate the confidentiality clauses of human subjects' research [11]. Typically, when researchers publish their results, only aggregate data on the entire sample or specific subsamples can be released.

Increasingly, the individual-level information collected as part of health-related research contains geographic identifiers. This can be relatively coarse in the form of the local jurisdiction (city or municipality) or postal code or much finer grained in the form of the exact street address. Some data collection protocols may also include the collection of coordinates using GPS units in the field. These geographic identifiers add value to the research in a number of different ways. First, if limited demographic and socioeconomic variables are available on the study subjects, their location can provide proxy variables. For example, it is very common to associate the study subjects with the demographic characteristics of the census enumeration unit they are located in. Second, the location of study subjects can provide insight into other variables which may be related to health outcomes. Examples include the time it takes to travel to the nearest health facility of interest, the distance to pollution sources, or the air/water/soil quality at their residential location.

Street addresses represent the most commonly used geographic identifiers for individual level data. Address information can be converted into locations on a map using a process known as geocoding [1, 12]. Geocoding can be accomplished using desktop GIS software or online mapping services. Automated geocoding methods can convert large address databases very quickly.

Geocoding is not error-free. Typically, a certain number of records do not geocode due to incomplete or incorrect information. Geocoded locations may also not be accurate due to incorrect reference information or errors in the geocoding process [1]. These errors, however, are relatively well understood and have received substantial attention in the literature [1, 13–20]. The datasets used for geocoding as well as the geocoding techniques themselves are also gradually improving [21, 22].

A review of articles published in recent volumes of some of the leading public health research journals reveals that geocoding is very widely used. In addition, several new health journals have emerged with a clear emphasis on the spatial dimensions of health, such as the* International Journal of Health Geographics* and* Spatial and Spatio-temporal Epidemiology*. This confirms that geocoding has become firmly established as an analytical tool in public health research [6].

## 3. Reverse Geocoding

**Figure 1:**
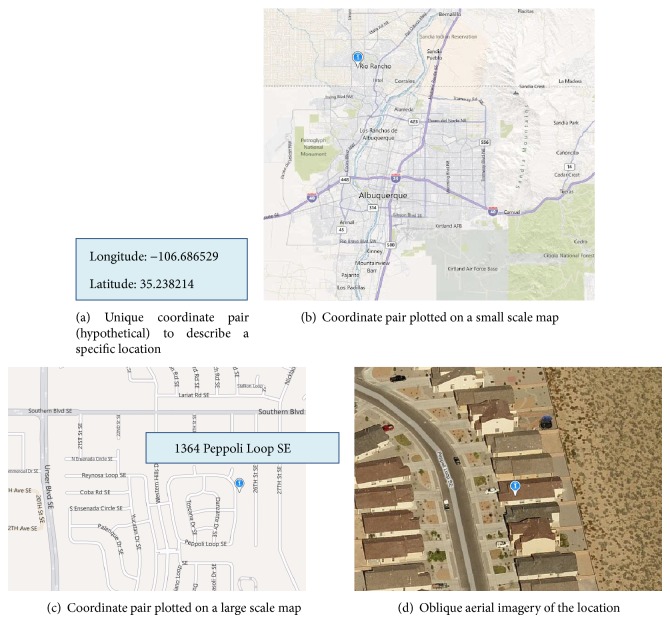
Disclosure of confidential information by publishing coordinates.  Figure 1(a) shows an example of a hypothetical set of coordinates. Plotting these on a small scale map (b) provides an approximate location (i.e., Rio Rancho). Zooming in using a large scale map (c) provides a very exact location, which can be used to identify the street address associated with the set of coordinates (e.g., 1364 Peppoli Loop SE). Aerial imagery (d) can be used to confirm the specific residence.

The widespread use of geocoding not only presents unprecedented opportunities for analysis, for example, [23–25]; it also presents challenges to preserving the confidentiality of public health datasets [2, 6, 26]. In short, the release of geographic information at the individual level can breach confidentiality. For example, publishing the street address of an individual makes it possible to look up the associated name(s) in directories and property databases. Publishing a location as coordinates (e.g., latitude/longitude) means that these can be plotted on a map and then associated with an address. Publishing a map in paper or digital form also means that the locations can be associated with an address.  Figure 1 illustrates an example where a published coordinate is published on a map to identify a specific residence.

**Figure 2:**
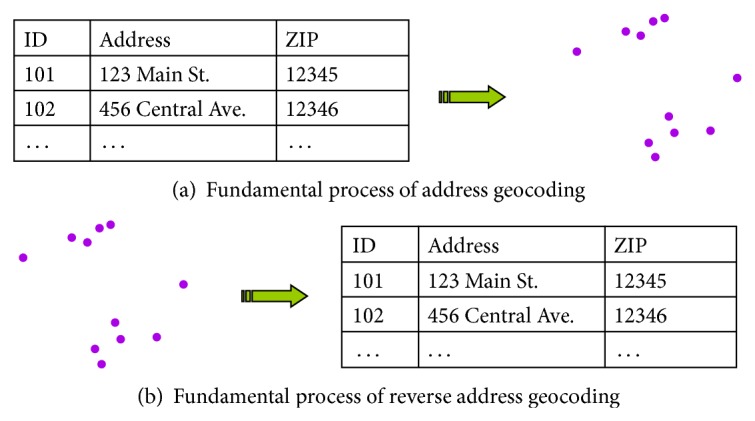
Geocoding and reverse geocoding. Geocoding (a) is the process of assigning locations (i.e., coordinates) to address information. A tabular dataset of addresses becomes a map. Reverse geocoding (b) literally puts this in reverse and converts mapped locations to addresses. Errors in the geocoding and reverse geocoding process may result in mismatched address information; that is, the addresses obtained using reverse geocoding may not be identical to those used in the original geocoding.

These techniques are collectively referred to as “reverse geocoding” [27–34]. Formally, reverse geocoding consists of determining the street address associated with a published location in paper or digital format. Reverse geocoding can lead to reidentification because the street address can then be associated with one or several individuals using common directories. Conceptually, reverse geocoding is like putting regular address geocoding in reverse, as illustrated in Figure 2.

Reidentification of individual addresses using reverse geocoding has been shown to be relatively easy and accurate. For example, [29] created a hypothetical map of geocoded patient addresses and were able to correctly identify 79% of the addresses using manual reverse geocoding techniques in GIS. The same authors employed a similar approach using semiautomated reverse geocoding based on image analysis and were able to correctly identify 26% of the addresses [35]. In another example, following Hurricane Katrina, a local newspaper published a map of mortality locations. Using a combination of GIS methods and field work, researchers were able to correctly identify the original residence for most of the locations in the published map [36]. More recently, a study of crime incidents in Vienna, Austria, determined the accuracy of reverse geocoding for several online mapping services [28]. Findings indicate that 68% of probable victims could be identified by name using online reverse geocoding and online address and telephone directories.

Current trends towards spatial data of greater detail and the availability of free online reverse geocoding tools increase the risk of reidentification [1]. For example, online geocoding services such as Google Maps and Microsoft's Bing Maps provide very accurate building-level geocoding and reverse geocoding as part of their (free) online mapping services. This has made accurate and relatively sophisticated “map-hacking” tools available to anybody with an internet connection and modest computer skills. The leading GIS software platform, ArcGIS by Esri, has also added a Reverse Geocoding tool to its standard set of data processing and analysis tools. This has further established reverse geocoding as a robust and standard GIS tool.

## 4. Benefits and Risks of Data Sharing

When trying to determine if and how locational information on individuals can be released, the following considerations need to balanced: (1) the need to protect confidentiality—this is part of an individual's right to privacy and most often a condition in the collection of the original data, (2) the desire to preserve the original pattern in the locations—this reflects the interest in trying to obtain useful information by utilizing individual level spatial data instead of aggregated data, (3) the usefulness of sharing data for the benefit of researchers and the benefit of the public at large. These considerations are conflicting in the sense that confidentiality is protected by maximizing changes to individual locations, while preserving the original pattern is accomplished by minimizing changes. The objective of any confidentiality protection method is to find a balance between reducing the risk for reidentification and preserving the properties of the original data.

These challenges in trying to balance the need for confidentiality with the potential benefits from providing researchers and others access to georeferenced individual health data have been widely recognized. For example, the National Research Council published a report in 2007 called “Putting People on the Map: Protecting Confidentiality with Linked Social-Spatial Data” [37]. The Panel on Confidentiality Issues Arising from the Integration of Remotely Sensed and Self-Identifying Data concluded that:

*“Recent research on technical approaches for reducing the risk of identification and breach of confidentiality has demonstrated promise for future success. At this time, however, no known technical strategy or combination of technical strategies for managing linked spatial-social data adequately resolves conflicts among the objectives of data linkage, open access, data quality, and confidentiality protection across datasets and data uses [37].”*

The present review documents some of the progress that has been made since the publication of this report and other studies with a similar message [38, 39]. Specifically, the review summarizes the state-of-the-art of geographic masking as one of the “technical approaches” referred to in the NRC report.

## 5. Confidentiality Protection Strategies

The simplest and most rigorous way to protect the confidentiality of study subjects is to simply not share any of the individual-level data collected as part of the research. For many datasets, this may be the best default option unless convincing arguments are available to release the data in some manner. One of the most practical and convincing arguments is that making data available has become a requirement of many funding agencies [40, 41].

One possible solution is to provide very restricted access to the individual-level data. This is the approach adopted by most cancer registries [10]. Individual-level cancer data records are collected and organized by cancer registries. Access to the individual records is restricted to researchers whose protocols have met the requirements of human subjects' review. Researchers are often restricted as to where they can use the data (sometimes on-site only) and what they are allowed to publish in terms of detailed results. This type of restricted access gives researchers the opportunity to work with the original, individual records, but subsequent releases of the data are strictly controlled. These detailed and institutionalized protocols are not very common for other types of health-related datasets.

**Figure 3:**
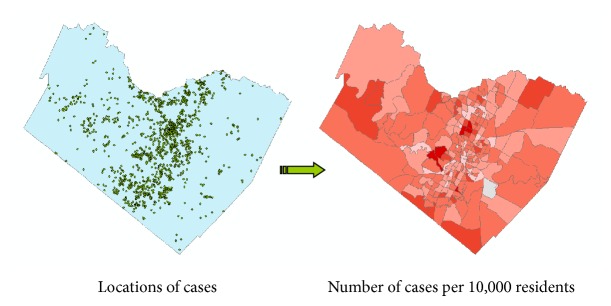
Spatial aggregation of individual cases using census enumeration units. Individual geocoded locations (left) are aggregated using census tracts (right). The count of the number of cases per census tract is used to determine relevant population-weighted indices, such as the number of cases per 10,000 residents. Determining incidence or disease rates, as opposed to raw counts, is one of the primary reasons for aggregation. As a secondary benefit, spatial aggregation greatly reduced the reidentification risk.

Another commonly used solution is to release the data in spatially aggregated form [9]. This is analogous to reporting summary data in tabular form for selected subsets of the original data. For individual-level geocoded data, aggregation is typically accomplished by combining individual locations within a meaningful spatial unit. This could consist of local or regional jurisdictions, such as cities, counties, or census enumeration units.  Figure 3 illustrates the basic process for spatial aggregation. To preserve confidentiality, only the aggregated dataset is published or shared.

For many applications, however, the release of spatially aggregated data is much less useful compared to having access to the individual locations [26]. Many spatial analytical techniques, such as general point-pattern analysis and cluster detection, are much less powerful or simply not possible using aggregated data.

Finally, an alternative solution is to modify the data in such a way that the risk for reidentification is greatly reduced without aggregating the data to coarser units of analysis. This includes altering the original locations in some systematic manner, also referred to as geographic masking.

## 6. Ensuring Confidentiality Using Geographic Masking

**Figure 4:**
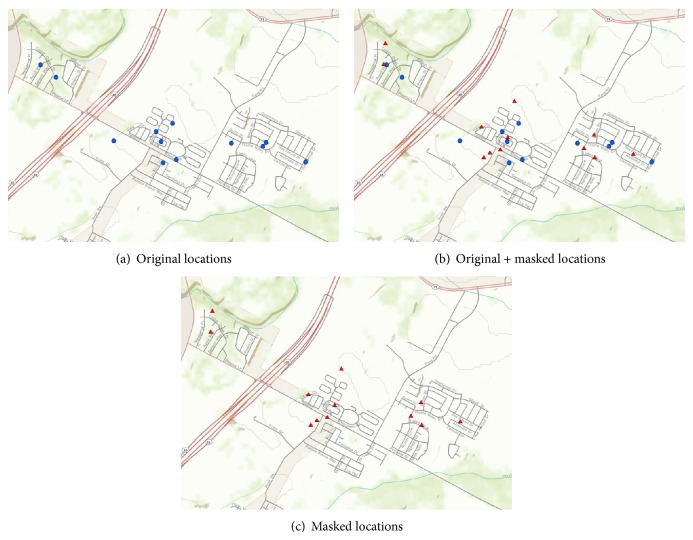
Conceptual illustration of geographic masking. A set of original locations (a) is created using address geocoding or field data collection using GPS. These locations correspond very closely to the residences of interest, although a certain amount of error might be present. For each location, a masked representation is created (b) by displacing the original location using one of several algorithms. Most algorithms include a certain degree of randomness in the displacement. The original locations are removed from the dataset, resulting in a set of masked locations (c) for publication and distribution purposes. The set of masked locations has the same number of observations as the set of original locations.

Geographic masking is the process of altering the coordinates of point location data to limit the risk of reidentification upon release of the data. In effect, the purpose of geographic masking is to make it much more difficult to accurately reverse geocode the released data.  Figure 4 illustrates the general concept of geographic masking.

The term geographic masking was first described in some detail in 1999 [26]. The term was introduced as an extension of masking techniques for nonspatial microdata [42, 43]. While geographic masking is the most widely accepted term, other terms have also been used, including “geomasking” [44–47], “jittering” [48, 49], and “dithering” [50]. The original description of geographic masking methods [26] included several different types of masking, including (1) affine transformations, which accomplish displacement using translations, changes in scale, and rotation, and (2) random perturbation, which accomplish displacement by adding a certain amount of random noise to the coordinates. The transformation approach has not been widely adopted, mostly because the new coordinates no longer have the same real-world context. For example, once a rotation or translation has been applied to a set of locations, it no longer makes sense to overlay these coordinates on top of other spatial data layers. As a result, geographic masking has become largely synonymous with applying random perturbation to coordinates.

Geographic masking is actively being used by public health researchers that use individual-level data. A number of studies were identified that met the following two conditions: (1) the article included a map with geocoded locations of individual-level health information and (2) specific mention was made that the geocoded locations were modified in some way for reasons of confidentiality (even if the term “geographic masking” was not used explicitly). The following section reviews the nature of these maps and the details reported on the geographic masking methods employed.

A study in Cape Code published maps with the location of residential addresses of patients diagnosed with cancer [48]. The geographic masking approach was described as “for confidentiality reasons, the points have been jittered [48].” A study in Churchill County, NV (USA), published maps with the location of residential address of childhood leukemia cases [51]. The geographic masking approach was described as “locations are enlarged and “jittered” to maintain confidentiality [51].” A study in England published maps of the locations of farms where bovine tuberculosis was found [52]. The geographic masking approach was described as “the map shows the location of each farm, jittered randomly within a circular disc of radius 5 km to preserve confidentiality [52].” A study in North Carolina published maps with the locations of the residential addresses of children screened for blood lead [24]. The geographic masking approach was described as “for publicly displayed maps… we randomly moved the actual location of the child within a fixed radial buffer, a technique known as jittering [24].” A study in Minnesota published maps with the locations of the residential addresses of persons diagnosed with cancer [53]. The geographic masking approach was described as “… plots the residential locations in this data, where we have added a random “jitter” to each in order to protect the confidentiality of the patients (and explaining why some of the cases appear to lie outside of the spatial domain) [53].” A study in Massachusetts (USA) published maps with the locations of the residential addresses of infants born to mothers living near a known PCB-contaminated Superfund site [54]. The geographic masking approach was described as “residence locations are jittered with 1% random noise to protect the confidentiality of participants [54].” A study in Perth, Western Australia (USA), published maps with the locations of the residential addresses of children who visited the emergency room with a principal diagnosis of asthma [54, 55]. The geographic masking approach was described as “case and control cases have been jittered [55].”

While these examples do not represent a comprehensive survey of all the published studies that employ geographic masking, they illustrate a number of characteristics. First, the term “jittering” is widely used instead of “geographic masking.” Although jittering is generally used to suggest some type of random perturbation, the examples vary in their use of the term. Second, a number of examples provide specifics on the nature of the geographic masking method, such as “jittered randomly within a circular disc of radius 5 km [52]” or “jittered with 1% random noise to protect confidentiality of participants [54].” Several other examples, however, simply state that locations have been altered without any further description.

## 7. Different Approaches to Geographic Masking

**Figure 5:**
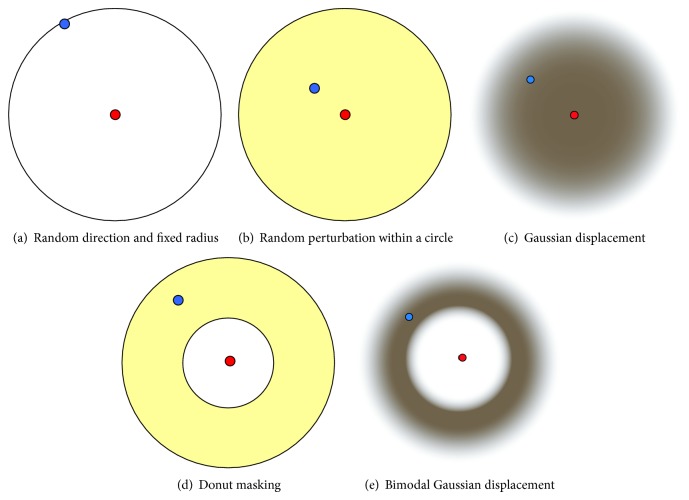
Graphical representation of common geographic masking techniques. The red dot indicates the original location and the blue dot one of the many possible masked locations.

A number of different geographic masking techniques have been developed over the years. All of these include some degree of randomization in order to reduce the risk of reidentification.  Figure 5 provides a visual representation of each of these methods.

*(1)  Random Direction and Fixed Radius.* Masked points are placed on a random location on a circle around the original location. Masked points are not placed inside the circle itself. 

*(2)  Random Perturbation within a Circle.* Masked locations are placed anywhere within a circular area around the original location. Since every location within the circle is equally likely, masked locations are more likely to be placed at larger distances compared to small distances. A variation on this technique is the use of random direction and random radius. In this technique, masked points are displaced using a vector with random direction and random radius. The radius is constrained by a maximum value. This effectively results in a circular area where masked locations can be placed, but the masked locations are as likely to be at large distances compared to small distances. These two techniques therefore only differ slightly in the probability of how close masked locations are placed to the original locations. 

*(3)  Gaussian Displacement.* The direction of displacement is random, but the distance follows a Gaussian distribution. The dispersion of the distribution can be varied based on other parameters of interest, such as local population density. 

*(4)  Donut Masking*. This technique is similar to random displacement within a circle, but a smaller internal circle is utilized within which displacement is not allowed. In effect, this sets a minimum and maximum level for the displacement. Masked locations are placed anywhere within the allowable area. A slightly different approach to donut masking is the use of a random direction and two random radii: one for maximum and one for minimum displacement. These two techniques only differ slightly in the probability of how close masked locations are placed to the original locations. Both approaches enforce a minimum amount of displacement. 

*(5)  Bimodal Gaussian Displacement.* This is a variation on the Gaussian masking technique, employing a bimodal Gaussian distribution for the random distance function. In effect, this approximates donut masking, but with a less uniform probability of placement.

While these methods are presented here as separate methods, several are slightly revised versions of each other. For example, donut masking and bimodal Gaussian displacement are very similar in terms of the general area where masked locations are placed relative to the original locations.

These five techniques have been described to varying degrees in the literature. Random direction and fixed radius have been used by [56]. Random perturbation within a circle has been studied by [26, 50, 56, 57]. Gaussian displacement has been studied by [57, 58]. Donut masking was originally proposed by [59] and has been studied in a number of more recent studies [44, 46, 47, 60]. Bimodal Gaussian displacement has been studied by [61]. These studies specifically focus on the development or testing of one or more masking methods. The earlier review of applications of geographic masking to real-world datasets has indicated that some studies do not mention the specific technique by name. Among those studies that do provide a description of the technique, the random perturbation is by far the most widely used. This suggests that the slightly more sophisticated methods that have received attention in the literature of geographic masking have so far not been adopted.

A number of other techniques have been mentioned in the literature, such as moving each location to the midpoint of the nearest street segment or to the nearest street intersection [62]. Technically speaking, however, these techniques are microspatial aggregation methods since several original locations may end up at the same “masked” location. While these methods warrant attention as an alternative to other methods of spatial aggregation, they have received very limited attention in the literature.

Determining the amount of displacement necessary to achieve confidentiality has been addressed by several of the studies on geographic masking [56], but no universal guidelines have emerged. However, it is widely agreed upon that the amount of displacement should be inversely proportional to the local population density [26, 47, 56, 58, 61]. For example, consider a residence in a rural area with a very low population density. It is quite possible that there are no other residences within 100 meters of this residence. A displacement of 100 meters would therefore not be very effective in reducing the reidentification disk. By contrast, a residence in a very densely populated urban area may be likely to have numerous other residences within 100 meters, and a displacement of 100 meters may be more than sufficient to substantially lower the reidentification risk. All masking techniques described above include at least one parameter that controls the overall magnitude of displacement, for example, the radius corresponding to the maximum displacement or the standard deviation for techniques employing a normal distribution. This parameter should be scaled inversely proportional to local population density (expressed as people per unit area). Instead of using the population density of census enumeration areas, several studies have proposed using the local density of residential addresses as a more reliable way to adjust the magnitude of displacement [44, 47, 63].

**Figure 6:**
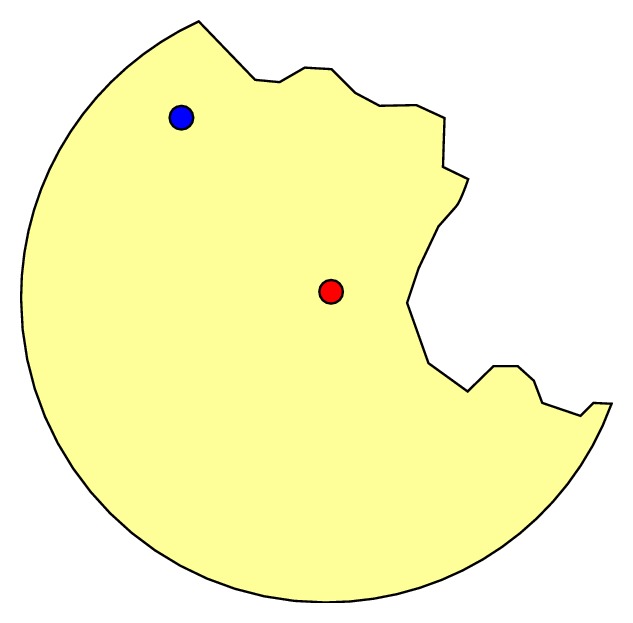
Example of geographic masking technique (i.e., random placement within a circle) using an additional spatial filter to constrain displacement. The red dot represents the original location; the yellow area represents all possible locations for the masked location; and the blue dot represents one possible masked location selected randomly. This filter can be used to avoid placement in areas where logically no population resides (such as water bodies or parks) or to limit displacement to a particular enumeration unit (such as the same census tract or postal code).

One variation on geographic masking is the use of additional spatial filters to ensure masked locations fall within predefined areas of interest. For example, displacement could be limited to a physical land base by excluding surface water bodies (e.g., oceans, bays, rivers, and lakes) to ensure that no masked locations appear in areas which are obviously uninhabited. Another potential use of such filters is to ensure masked locations stay within the same enumeration units (e.g., census block group, postal code) as the original location. The use of such additional spatial filters is illustrated in Figure 6.

While conceptually relatively simple, no studies on geographic masking have specifically addressed the use of such additional spatial filters. It is therefore not known, for example, to what extent their use increases the risk for reidentification.

## 8. Effectiveness of Geographic Masking in Preserving Confidentiality

One critical aspect in evaluating the effectiveness of geographic masking is to determine how the masking algorithm has reduced the risk of reidentification. In other words, what is the probability of discovery of the masked dataset? This is critical for finding the much-desired balance between protecting confidentiality and maintaining data utility.

Many early studies on geographic masking essentially postulated that a “substantial” displacement of the original point location would suffice to preserve confidentiality [56, 64]. More recently, determining the nature or magnitude of displacement required to effectively accomplish this has started to receive more attention [44, 46, 61, 65].

Several approaches have been developed to determine the degree of confidentiality provided by specific geographic masking techniques. The most widely embraced approach that has started to receive interest in recent years employs the concept of “spatial *k*-anonymity.” This extends the concept of “*k*-anonymity,” which provides a quantitative estimate of the probability of discovery for tabular data [66–70]. Traditional *k*-anonymity implies that data for a particular individual will only be released if there is a minimum of *k* − 1 individuals with the same combination of characteristics. When a particular value for *k* is determined, data tables can be empirically examined to ensure that the expectation for *k*-anonymity is met.

**Figure 7:**
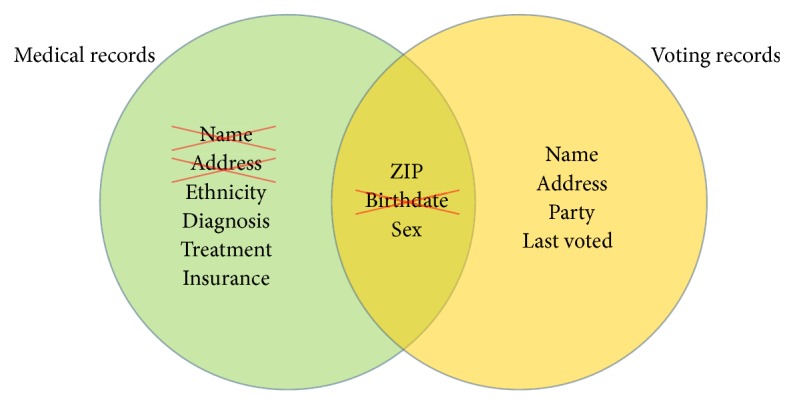
Illustration of the *k*-anonymity concept using record linkage. Medical records contain a number of different fields which are removed to protect confidentiality, including name and address. When combined with voting records, however, it becomes possible to uniquely identify individuals in the medical records by combining fields for ZIP code, birthday, and sex. The *k*-anonymity provided by the released data is unacceptably low. By removing the field for birthdate (or replacing it with birth year), the *k*-anonymity is substantially increased and may reach acceptable levels. The concept of *k*-anonymity provides a quantitative measure of confidentiality protection. More specifically, it is a number that can be calculated for each subset of the data. For the example of medical record and voting records, values for *k*-anonymity can be calculated prior to release for all combination of ZIP code and sex or any other field of interest. Adapted from [66].

The concept of *k*-anonymity is best illustrated with an example, adapted from [66] and shown in Figure 7. Consider a set of health-related records with personal identifiers such as name, birthdate, sex, ethnicity, street address, and ZIP code, in addition to health-related data such as diagnosis, treatment, and insurance. To protect confidentiality, individual identifiers need to be removed from the data prior to release, including name and address. While this may appear to be sufficient to protect confidentiality, consider a second set of records consisting of publicly available voting records. In many jurisdictions these records include the individual's name, birthdate, sex, street address, and ZIP code, in addition to voting-related data such as party affiliation and the nature of participation in the last election. The voting records can be used to reidentify the individuals in the anonymized health records. In this particular example, the combination of ZIP code, birthdate, and gender in most cases will uniquely identify a single individual. The value for *k* would be 1, which is of course unacceptable. A possible solution is to replace the exact birthdate with the birth year, although in some cases this may not be sufficient. For an actual set of data files, empirical values for *k* can be determined to see the effects of specific anonymization techniques on the risk of reidentification.

The *k*-anonymity concept can be expanded to include geographic identifiers. Spatial *k*-anonymity is an emerging concept that has started to receive some attention for testing and comparing geographic masking techniques [65, 71, 72]. Similar to *k*-anonymity for nonspatial data, spatial *k*-anonymity provides a quantitative estimate of the probability of discovery, but now considers reverse geocoding instead of database record linkage as the primary mechanism for reidentification.

Spatial *k*-anonymity has been applied fairly extensively to the protection of privacy in location-based services [71, 73–75]. In the context of individual residential locations, however, spatial *k*-anonymity has not been well developed. In general, determining an estimate for spatial *k*-anonymity for residential locations relies on a comparison between the amount of displacement of a location introduced by masking and the density of the local population of interest. A relatively large displacement in an area of high population density would provide a high degree of spatial *k*-anonymity. One proposed approach to implement this logic is referred to as the “*n*th nearest neighbor number” method, that is, the number of potential residential locations which are in closer proximity to the masked location than the original location [44, 47, 63]. This approach employs the empirically observed distribution of actual residential locations. The *n*th nearest neighbor values can be used to provide an empirical estimate of spatial *k*-anonymity, similar to the example of database record linkage discussed previously. One shortcoming of this approach is that it relies upon the availability of high resolution residential address points or buildings. A variation on this approach has been developed using population density for census enumeration areas instead of the distribution of actual residential locations [61]. While there have been few studies using spatial *k*-anonymity to examine the reidentification risk associated with masked datasets, in a typical setting larger displacements have been shown to result in the highest values for spatial *k*-anonymity [44, 47, 61], as expected.

Given the nature of geographic masking, any type or amount of displacement or perturbation of the original locations will still allow for the theoretical possibility that the masked location is in relatively close proximity to the “true” location. However, the actual distance is not as important as the probability of discovery, which is more effectively characterized by an analysis based on spatial *k*-anonymity. Therefore, if a location is displaced by a substantial distance, but the spatial *k*-anonymity value is still very low, the probability of discovery is still substantial. This could be the case in a low density rural area where even a substantial displacement may not provide adequate protection of confidentiality.

A standard for confidentiality protection when publishing individual-level locations does not exist at the present time. However, as a general guideline for researchers, such a standard could be based on achieving a high level of spatial *k*-anonymity. The basic question for research into geographic masking techniques should therefore be which geographic masking parameters are necessary to obtain high values for spatial *k*-anonymity? More specifically, which geographic masking parameters are necessary to provide a specified minimum level of *k*-anonymity for a given dataset? The use of quantifiable measure of the probability of discovery in the form of an index for spatial *k*-anonymity greatly facilitates this line of reasoning. For example, for a specific case-study of emergency department visits in the area of Boston, MA (USA), an average displacement of 0.25 km was found to result in a spatial *k*-anonymity value of 20 or higher for 99% of the original locations [61].

There has been surprisingly little research comparing the effectiveness of different geographic masking techniques. Most studies have examined only a single method in the context of a specific scenario. Despite this lack of comparative studies, there appears to be general agreement that donut masking and bimodal Gaussian displacement are preferred over other techniques since they enforce a minimum amount of displacement. Random perturbation within a circle and simple Gaussian displacement may result in masked locations which are very close to the original locations. For simple Gaussian displacement these nearby locations are in fact the most likely. This is undesirable since it presents a high risk of reidentification through reverse geocoding. While this argument is supported by logic, few studies have provided empirical analysis to demonstrate these potential advantages [46]. The lack of comparative analysis of masking techniques provides a clear indication for desirable future research directions.

## 9. Multiple Releases of Masked Data and Disclosure of Masking Methods

Confidentiality may be breached by releasing multiple versions of the same masked datasets [57]. For example, an agency responsible for releasing location information may rerun the geographic masking algorithm with every request for a particular dataset to ensure that each release is unique. If such multiple releases were made available, these could be combined to aid in the reidentification of the original locations. Multiple releases at least in theory make it possible to reverse engineer the masking algorithm used to create the masked datasets. Therefore, even if the masking algorithm itself is not released, multiple releases of the data may present an increased reidentification risk.

Different masking techniques will vary in their robustness to this form of reidentification. However, most techniques in their basic form are symmetrical (i.e., displacement direction is random and displacement distance does not depend on direction). As a result, the average location of a large number of masked locations will start to approximate the original location. Additional perturbation may be introduced if separate masked locations are in close proximity to each other and can therefore not be distinguished in multiple versions of the masked data sets. Even in this scenario, however, averaged locations of multiple locations in multiple masked datasets will provide insights into the masking methods, which in itself will lead to increased disclosure risk. While this effect has been recognized in the literature on geographic masking [57, 58], very limited empirical testing has been carried out.

One additional aspect to consider is the release of the specific geographic masking technique together with the masked dataset. Conceivably, knowledge of the algorithm provides additional knowledge to identify the original location. Similar to the way geocoded locations can be identified using reverse geocoding, masked locations could be identified using “reverse geographic masking.” This has received some attention in the literature [57], but has been limited in terms of datasets and masking methods. It is expected that different masking techniques will vary in their robustness to this form of reidentification. For example, the random direction and fixed radius method are not expected to be very robust in this regard.

## 10. Effects of Masking on Spatial-Analytic Methods

Typically the most compelling reason to release individual-level health datasets in some form is that they provide more useful information than the summarized or spatially aggregated versions of the same data. Many types of analysis are only possible using the individual points. It is therefore critical to determine to what extent the properties of these datasets are preserved by geographic masking. If geographic masking results in a point pattern whose properties do not closely resemble those of the original point locations, then the individual-level dataset is of much lower analytical value.

Research on the effects of geographic masking on the spatial-analytic properties of a set of location is essential in order to determine whether masking technique strike a meaningful balance between the protection of confidentiality and the ability to derive relevant spatial relationships. What follows is a summary of the studies to date on this subject. A study in Franklin County, Ohio, Kwan et al. [56] used the residential addresses of 541 deaths due to lung cancer to examine the effects of two different masking techniques: random direction with a fixed radius and random placement within a circle, using different radii for both methods. Effects of masking were determined by using kernel density estimation and the cross *K*-function. Findings indicated a consistent tradeoff between the amount of perturbation and the accuracy of the analytical results [56]. A study using artificial clusters of point locations masked using bimodal Gaussian displacement examined the robustness of cluster detection using SaTScan [61]. Results showed a gradual decrease in cluster detection sensitivity and specificity with an increase in the average displacement distance. A study on household travel surveys employed donut masking for a set of selected households and examined the influence of masking on measures of the build environment [60]. Results showed a gradual reduction in the utility of these measures with larger displacement distances. A study on the location of burglaries determined the effect of masking on measures of spatial point patterns (Nearest Neighbor Index) and on measures of clustering after spatial aggregation (Moran's I) [47]. Results indicated very minor effects of geographic masking for displacements of up to 250 m. A study using masked versions of simulated points determined the robustness of kernel density estimation [50] and found a strong influence of the search radius (or bandwidth). Displacements greater than 1/5th of the search radius were found to result in substantial differences in the final results.

The literature on the effects of geographic masking on the robustness of spatial-analytic technique is relatively limited. However, lessons can be learned from the much larger body of the literature on the effects of positional errors in geocoding on spatial analyses [1, 13–16, 18, 20, 76–82]. While geographic masking is not a type of geocoding error, the net effect on spatial analysis is very similar: locations are displaced in a systematic manner and this introduces a certain amount of error in the spatial analytic procedures using these locations as input. The primary difference is that the displacements in geographic masking falls within a very specific range and often follows a uniform or normal distribution, while positional errors in geocoding follow a log-normal distribution [16, 83]. This means that a set of locations obtained using geocoding typically contains a large proportion of locations with a relatively small error (up to 100 meters or thereabouts) and a much smaller but not insignificant proportion of locations with a much larger error (up to several hundred meters or even kilometers). Despite this difference, the geocoding literature provides some useful insights into the effects of location displacement on the outcome of spatial analysis. In general, this research suggests that the effects are highly dependent on the type of analysis method and the specific scale of the analysis. For example, research on kernel density analysis suggests that the robustness of results is highly dependent on the search radius employed in constructing the kernel [15] with very small values for the radius producing very unreliable results. Similarly, concordance with census enumeration units is dependent on the typical size of the polygons being used, with smaller units resulting in larger errors in the analysis [15, 84].

While most studies have examined the effect of geographic masking using very specific spatial-analytical procedures, other less technical approaches have also been used. For example, [62, 64] have used human study subjects to identify the effect of masking techniques on the visual impact of point patterns.

## 11. Alternatives to Masking

Geographic masking methods have been under development for over 10 years. Despite the development of several different masking techniques, there is no general consensus on which technique is most suited for a particular task. Based on the progress in developing and testing masking techniques, it is unclear whether advances in geographic masking will lead to the widespread adoption and recommendation of a particular set of techniques. It is therefore worthwhile to consider what alternatives are available. These alternatives fall into a number of categories.

One approach to more traditional geographic masking is the use of more complex spatial manipulations of the data. Proposed approaches include spatial smoothing [85], multiple imputation [86], and linear programming [65]. While these methods manipulate the original locations using spatial analytic methods, they do not fall under what are commonly referred to as geographic masking techniques.

A more radical alternative to geographic masking is the use of synthetic data. In this approach, a dataset is created which has properties that are very similar to that of the original data, but the identities of all individuals have been modified. This approach has been successfully developed for tabular datasets [87].

Software agents present another alternative. In this approach, software is used to provide controlled access to original individual data records without releasing identifiable details [88]. Analysis results are returned based on the individual records. This approach does not suffer from the limitations presented by releasing spatially aggregated data. There is a concern that certain properties of the original data could be inferred from the analysis results, but in general the risk of reidentification is much lower compared to the release of individual-level masked datasets [88]. While very promising in concept, the use of software agents to handle confidential health datasets is not very widespread, in part, because of the challenges related to establishing the secure computer infrastructure to implement the approach.

Yet another alternative is to employ flexible aggregation methods which are much finer than traditional census units, but which do not reveal exact individual locations [89]. Such flexible aggregation methods provide an easily quantifiable measure for the risk of reidentification, while at the same time minimizing the degree of aggregation to limit the decrease in the utility of the data.

While a number of alternatives to geographic masking have emerged, there have been no comparative studies to examine the relative strengths of various approaches for a specific application. As a result, there is currently no clear guidance on when geographic masking method should be employed and when alternatives should be considered.

## 12. Conclusions

The growing body of knowledge on geographic masking indicates that it is possible to provide a quantitative estimate of the degree of confidentiality provided by a specific masking technique for a given study area. It is also possible to quantitatively determine the effects of geographic masking on the robustness of specific analytic techniques. This suggests that finding a balance between confidentiality protection and data utility is technically possible for a given scenario. Despite this recent progress, at the present time there is no universally accepted or endorsed geographic masking method. Research and funding agencies do not provide any guidance on what masking methods to use or how to use them.

This gap can likely be attributed to a number of factors. First, while awareness of confidentiality concerns is high, spatial literacy among most health researchers is not. Geocoding and basic spatial analysis techniques have become widely used in public health research, but topics such as reverse geocoding, geographic masking, and spatial *k*-anonymity have not yet become part of the vocabulary of mainstream public health research. Second, the number of studies on geographic masking is still relatively small and the research community has not presented a very strong case for a particular set of methods that would be effective for a range of different scenarios. Third and perhaps most important, it is not clear that geographic masking presents the best alternative among several approaches to protect confidentiality while providing controlled access to individual-level data for analysis and surveillance purposes. While geographic masking clearly holds promise, it is limited in what can be accomplished technically and alternative approaches may prove more effective at achieving the same general goals for specific applications.

This suggests a number of different avenues for future research. First, research on geographic masking is clearly in its early stages, and more work is needed to compare existing approaches and to develop new ones. Second, technical guidelines are needed on the use of geographic masking. While decisions on if and how to release georeferenced individual-level health data are obviously not based on technical criteria alone, a better understanding of the possibilities and limitations of geographic masking should contribute to more informed decisions. Third, several alternatives to geographic masking have been developed and research is needed to compare the strengths and weaknesses of these approaches relative to more established masking techniques.

Researchers on the other hand are publishing maps using geographic masking of confidential locations, in the absence of clear guidelines on how to best accomplish this. Any researcher publishing such maps is advised to become very familiar with the different techniques available and their associated reidentification risks.
